# APSIC dental infection prevention and control (IPC) guidelines

**DOI:** 10.1186/s13756-023-01252-w

**Published:** 2023-05-30

**Authors:** M. L. Ling, P. Ching, J. Cheng, L. Lang, S. Liberali, P. Poon, Y. Shin, C. Sim

**Affiliations:** 1 Singapore General Hospital, Outram Road, Singapore, 169403 Singapore; 2 The University of Hong Kong, Hong Kong, China; 3 Taipei Medical University, Taipei, Taiwan; 4 National Healthcare Group Polyclinics, Singapore, Singapore; 5 University of Adelaide, Adelaide, Australia; 6 Department of Health, Hong Kong, China; 7 Seoul National University, School of Dentistry, Seoul, South Korea; 8 National Dental Centre, Singapore, Singapore

**Keywords:** Infection prevention, Infection control, Dental

## Abstract

**Background:**

The Asia Pacific Society of Infection Control launched the Infection Prevention and Control Guidelines in July 2022. This document describes the guidelines and recommendations for safe practices in dental setting. It aims to highlight practical recommendations in a concise format designed to assist dental facilities at Asia Pacific region in achieving high standards in infection prevention and control practices, staff and patient safety.

**Method:**

The guidelines were developed by an appointed workgroup comprising experts in the Asia Pacific region, following reviews of previously published international guidelines and recommendations relevant to each section.

**Results:**

It recommends standard precautions as a minimal set of preventive measures to protect staff and prevent cross transmission. Surgical aseptic technique is recommended when procedures are technically complex and longer in duration. Only trained staff are eligible to conduct reprocessing of dental instruments. The design, layout of the dental facility are important factors for successful infection prevention. The facility should also have a Pandemic Preparedness Plan.

**Conclusions:**

Dental facilities should aim for excellence in infection prevention and control practices as this is part of patient safety. The guidelines that come with a checklist help dental facilities to identify gaps for improvement to reach this goal.

## Introduction

COVID-19 pandemic had recently highlighted the of transmission mechanism of SARS-CoV-2 through respiratory droplets and aerosols [1, 2]. Attention to infection prevention and control (IPC) practices in dental units is critical to stop cross-infection. The intent of this document is to highlight IPC recommendations in a concise format designed to assist dental facilities at Asia Pacific region in achieving high standards in IPC practices to minimize cross infection between the dental care practitioner and the patient. Particularly in dental treatment settings, practitioners are often exposed to contacts with patients, blood and bodily fluids. Additionally, the use of sharp instruments significantly increases the risk of exposure to infection, and as dental treatment is a type of outpatient, invasive treatment, effective controls for outpatient-derived infections are essential. This document is a summary of the APSIC Dental Infection Prevention and Control (IPC) Guidelines developed by the Asia Pacific Society of Infection Control (APSIC) to give the user an overview of its content. The full APSIC Dental Infection Prevention and Control (IPC) Guidelines available at https://apsic-apac.org should be read and used as reference to guide practice.

### Guidelines development workgroup composition

APSIC convened experts in infection prevention and control and dentistry from Asia Pacific region to develop the APSIC Dental Infection Prevention and Control (IPC) Guidelines. The members of this workgroup are the authors of this paper.

### Literature review and analysis

For the APSIC guideline, the workgroup reviewed previously published international guidelines and recommendations relevant to each section and performed computerized literature searches using PubMed.

### Process

The workgroup met on 2 occasions as well as discussed via email correspondences to complete the development of the guideline. Criteria for grading the strength of recommendation and quality of evidence are described in Table 1. The draft was then submitted to two external reviewers, APSIC executive committee and national infection prevention and control societies in Asia Pacific. Comments obtained were then reviewed by the workgroup for necessary edits, following which the final copy was circulated for approval and endorsement by the APSIC executive committee and national societies from the Asia Pacific region.

**Table 1:** Categories for strength of each recommendation

Categories for strength of each recommendation	
Category	Definition
A	Good evidence to support a recommendation for use
B	Moderate evidence to support a recommendation for use
C	Insufficient evidence to support a recommendations for or against use
D	Moderate evidence to support a recommendations against use
E	Good evidence to support a recommendation against use

Categories for quality of evidence on which recommendations are made	
Grade	Definition
I	Evidence from at least one properly randomized, controlled trial
II	Evidence from at least one well-designed clinical trial without randomization, from cohort or case-controlled analytic studies, preferably from more than one centre, from multiple time series, or from dramatic results in uncontrolled experiments
III	Evidence from opinions of respected authorities on the basis of clinical experience, descriptive studies, or reports of expert committees

### General IPC measures [3–5]

In the dental care settings, various transmission mechanisms are possible—contact with body-derived substances and contaminated environments, droplet transmission, and airborne transmission by aerosols, etc. Effective precautions are essential since both patients and staff are at risk of exposure to blood-borne pathogens while performing health care or nursing. Patients suspected of having infections transmitted by airborne route should be rescheduled until the period of communicability is over.

In emergency situations and procedures are unavoidable, the patient should preferably be seen as the last patient of the day, with appropriate barrier precautions used and staff assisting in the dental treatment must be aware of their immune status for the relevant infectious disease of the patient. The use of dental dam, where possible, for restorative work is recommended to reduce exposure of dental practitioners and clinical support staff to potentially infected aerosols. When treating these patients, it would also be prudent for clinical staff to wear well-fitted masks or respirators with high filtration capabilities such as the N95 respirator. It would also be prudent to use pre-procedural mouth rinses and appropriate disinfectant for surface cleaning and disinfection at the end of the appointment.

In general, the following recommendations for routine work will include the following:

1. Standard precautions are to be complied with by all dental staff. [A1I]
2. Transmission-based precautions (airborne, droplet and contact precautions) should be practised in addition to standard precautions where appropriate. [AII]
3. Airborne precautions include use of a special ventilated room with negative pressure and staff must wear N95 or FFP2/FFP3 respirators. [BI]
4. Droplet precautions include staff wearing a surgical mask on entering the room. [BI]
5. Contact precautions include the use of disposable gloves and gown. [BI]
6. Reschedule patients with pulmonary tuberculosis, chicken pox and measles. [BII]

### Asepsis and surgical management [6–10]

Aseptic technique aims to prevent the introduction of micro-organisms from hands, surfaces and equipment to a susceptible sterile site. Surgical aseptic technique is demanded when procedures are technically complex, and longer in duration. The concept of a main critical aseptic field is considered and hence, sterile gloves and protective barriers (e.g. drapes) are required. A surgical hand scrub is required prior to any aseptic task or procedure.

Recommendations on asepsis and surgical management include:

1. The principles of IPC and standard aseptic technique must be applied to all dental procedures, specifically those which are technically simple and short in duration (approximately < 20 min). [AI1]
2. The principles of IPC and surgical aseptic technique must be applied to all surgical dental procedures, particularly those where there is a planned penetration of the oral mucosa. [AI1]
3. Effective hand hygiene is an essential part of aseptic technique. [A1]
4. A surgical hand scrub using an antimicrobial handwashing solution, or an alcohol based hand rub (ABHR) approved for surgical hand decontamination, is required for surgical aseptic technique [A1]
5. Sterile gloves must be used for surgical aseptic technique [A1]
6. An aseptic field is necessary to provide a controlled aseptic working space to help maintain the integrity of asepsis during surgical procedures. [AI1]

### Dental environment [3, 11–15]

The facility is divided into two zones: clean and contaminated zones, where the clean zones are where there is no patient care activities e.g. staff room, office area, waiting and reception areas, storage supply area and sterilized instruments and equipment; and the contaminated zones are determined by what is touched and where the droplets, splash or spatter has spread. Clinical contact surfaces in the contaminated zone not barrier protected must be cleaned after each patient.

Routine cleaning of the clinical area is necessary to maintain safe environment because dust soil and microbes on the environment surfaces can transmit infection.

The environment should be clean, free from dust, dirt and body fluid stains and spillages. Compliance with safe care and safe working environment is the key to quality care. The use of audit check list to conduct regular audits help identify discrepancies between standards and the actual practices among the team.

Management of medical and related waste (also referred to as contaminated waste) must conform to national and institution regulations. It is recommended that the dental facility develop a waste management program according to the national and institutional regulation and guide.

Most dental unit waterlines contain biofilm, which acts as a reservoir of microbial contamination. It is recommended that dental unit waterline systems must be regularly maintained, via water treatment and monitoring, and performed according to the manufacturer’s instruction.

Recommendations for safe dental environment include:

1. Establish policies and procedures for routine cleaning and disinfection of the environmental surfaces in dental healthcare settings. [AIII]
  - If surface barriers are used to protect clinical contact surfaces (e.g., switches on `dental chairs, computer equipment) change surface barriers between patients.
  - Clean and disinfect clinical contact surfaces that are not barrier-protected with approved hospital grade disinfectant at the start of the day and after each patient. [BIII]
2. Select EPA-registered disinfectants or detergents with label claims for use in health care settings. [AIII]
3. Follow manufacturer instructions for use of cleaners and EPA-registered disinfectants (e.g. amount, dilution, contact time, safe use, disposal). [BIII]
4. Follow national and institutional regulation on managing different types of waste. [BII]
5. Use water that meets the CDC recommended limit for dental procedural water (i.e., ≤ 500 CFU/mL of heterotrophic water bacteria) for routine dental treatment output water. Adopt appropriate infection control procedures for dental unit waterlines. These include flushing dental unit waterline, use of germicidal product, biofilm prevention and monitoring of water quality from the dental unit waterline. [AII]
6. Use only sterile saline or sterile water as a coolant/irrigant when performing surgical procedures. [AII]

### Special procedures and IPC issues [16–19]

Potential occupational exposures at the dental facility may occur arising from grinding, polishing or other aerosol generating procedures at laboratories, surgeries, etc. Standard precautions including appropriate environment/equipment hygiene would help ensure all health activities are carried out in a safe and healthy environment for both dental health care professionals (DHCPs) and patients.

Recommendations for special procedures include:

1. All impressions and appliances should be thoroughly cleaned and rinsed of all debris before being handled in the on-site laboratory or sent to an off-site laboratory. [BII]
2. The dental on-site laboratory staff should wear appropriate PPE (mask, gloves and protective eyewear) to perform disinfection. [AII]
3. Heat-tolerant items used in the mouth must be cleaned and heat sterilized before being used on another patient. [AII]
4. Environmental surfaces should be barrier-protected or cleaned and disinfected with low-level disinfectants [AII]
5. Appliances and prosthesis delivered to the patient should be free of contamination. New and old dentures should be disinfected and rinsed by treated water (tap water that is safe for drinking as stipulated by national regulation). [AII]
6. In radiography room, when the surface is visibly contaminated with blood or saliva, intermediate level disinfectant should be used. [AII]
7. Radiography equipment should be cleaned and disinfected with low level disinfectant after each patient use or should be protected with surface barriers. [BII]
8. In heavy aerosol environment, high volume evacuation must be used as routine practices, and preventable by routine practices [BII]
9. Critical OPD surgery should have pre-procedural mouth rinses for patients to decrease the number of microorganisms during invasive dental procedures. [AII]

### Instruments handling and reprocessing [20–23]

The dental instrument reprocessing cycle includes the vital steps of cleaning and disinfection, inspection, packaging, sterilization, documentation and before they are reused on the next patient. These are assigned to DHCPs with training in the required reprocessing steps to ensure reprocessing results in a device that can be safely used for patient care, including the appropriate use of PPE necessary for safe handling of contaminated equipment.

Pre-cleaning is strongly recommended before disinfection to improve the safety and effectiveness of instrument reprocessing. It ensures the removal of organic remnants that the microbes are embedded in. If this organic layer is not removed properly, it can shield the microorganisms and potentially compromise the disinfection or sterilization process. If cleaning is delayed, the soiled instruments can be kept in liquid solution which could be any proprietary product for pre-cleaning. Drying out of the uncleaned debris will make subsequent cleaning more difficult. Separation of dirty and clean zones must be clearly demarcated to ensure no mixing of contaminated instruments from cleaned/disinfected instruments before sterilization.

Cleaning verification by users must be performed to assess level of cleanliness of instrument surfaces. This can be done by mainly visual inspection with the aid of lighted magnifying lens if needed. Other verification methods (e.g. ATP, protein residue, etc.) if deemed necessary can also be used.

Regular monitoring of the sterilization cycle is necessary to ensure the sterility of reprocessed instruments. Where there is a sterilization failure, a product recall must be activated and the use of the sterilizer stopped immediately. The process of sterilization must be reviewed to rule out the possibility of operator error. If there is any procedural error/failure identified, this should be rectified and subsequently retest the sterilizer, performing the physical, biological and chemical monitoring.

Recommendations on instrument handling and reprocessing include:

1. Proper cleaning, disinfection and sterilisation processes must be clearly stated in all dental clinics and preferably be carried out by trained dental healthcare professionals. [BII]
2. Proper sterilisation of dental handpieces and all dental instruments is important and should follow the manufacturer instructions. It is important that proper sterilisation is performed to prevent transmission of microorganisms. [AII]

### Incident and outbreak management [24, 25]

Exposure to blood or saliva by percutaneous injury with sharp injury is the greatest risk for acquiring a blood-borne pathogen in the dental health-care setting. The dental facility should have a post-exposure management protocol where staff is immediately evaluated to assess the potential to transmit an infectious disease. An outbreak, if any, will need to be investigated and risk assessment done to evaluate possible environmental exposures.

Recommendations on managing incidents and outbreaks include:

1. There should be a process of notification of supervisors, senior management and IPC. [AIII]
2. A procedure should be established for the recall of improperly reprocessed medical equipment/devices. [AIII]

### Education and training [26, 27]

Infection prevention education and training with ongoing program for DHCPs are critical for ensuring that infection prevention policies and procedures are understood and followed. The supervisor needs to ensure:

- IPC education and training, such as hand hygiene, use of PPE, appropriate to their position and responsibilities is provided upon hire, at least annually, and whenever new equipment or processes are introduced.
- Education on the basic principles and practices for preventing the spread of infections should be provided to all DHCPs.
- Training should include both DHCP safety (e.g., OSHA blood-borne pathogens training) and patient safety (e.g. dental instrument sterilization and disinfection training course)
- Regular refresher training is also appropriate to ensure the necessary infection control measures are being complied with and understood
- Regular continuing education is required and be supported, as well as encouraged
- There are regular documented internal audits to assess the competency of staff involved in IPC procedures
- IPC policies are reviewed by all staff members and updated at least annually.

Recommendations on education and training include:

1. There is a written policy regarding immunizing DHCP, with immunization program. [CI]
2. Develop and maintain regularly updated immunization/health records for dental staff. [BI]
3. Provide job- or task-specific infection prevention education and training to all DHCPs. [BI]
4. Provide training during orientation and at regular intervals (e.g., annually). [BI]
5. Maintain training records according to state and national requirements. (IB)

### Pandemic preparedness [11]

Following the 2009 influenza H1N1 and the 2020/21 COVID-19 pandemics, it is clear that dental care services and clinics should have a pandemic preparedness plan. Such plans shall be reviewed periodically with reference to the most updated scientific evidence and the local health authority.

Patients suspected with a pandemic associated infection should be isolated in separate room as far as possible. Consider postponing elective procedures and non-urgent patient visits when client reports signs and symptoms of the pandemic infection and positive history of epidemiological clues (acronym TOCC), i.e.

- Travel to areas with known epidemic of potential concern (EPC) within the known or suspected incubation period
- Possible Occupational exposure to pathogens of potential concern (O)
- Unprotected Contact with those with the EPC within the known or suspected incubation period
- Being part of a rapidly spreading Cluster of patients with the infection of unknown cause, including exposure to household members with the EPC.

Recommendations on pandemic preparedness include:

1. Dental care services clinics should have a pandemic preparedness plan with reference to the local health authority. [BII]
2. Early identification through client triage process in pandemic situations is key to prevent spread of infectious disease in clinic settings. [AII]
3. Consider if elective procedures and non-urgent patient visits be postponed when patient reports signs and symptoms of the pandemic infection and positive TOCC history. [BII]
4. Avoid AGP as far as possible. If unavoidable, AGPs should be carried out in negative pressured rooms. [BII]
5. Use fourhanded dentistry, high evacuation suction and dental dams to minimize droplet spatter and aerosols. [AII]
6. DHCP should wear appropriate PPE for standard precautions and transmission base precautions according to pandemic guideline recommendations. [BII]
7. Practices should ensure physical distancing. Good hand and respiratory hygiene measures are followed at all times throughout the practice. [BII]

### Design specifications [28, 29]

The design, layout of dental surgery and treatment areas are important factors for successful infection prevention. Work areas should be well lit and ventilated with sufficient uncluttered and easily cleaned bench space to contain dental equipment. There must be a defined area for clean and contaminated zones in the dental operatory and instrument reprocessing rooms. All dental staff must understand the purpose of and requirements within each zone, and adhere to the protocols.

1. There should be clearly defined clean and contaminated zones. [AII]
2. It is highly recommended that the operatory be in single room [BII]
3. Alcohol-based hand rub (ABHR) dispensers should be installed at every operatory at the point of care to facilitate easy access to hand hygiene. [AII]
4. The reprocessing area must be divided into distinct areas for:
  Receiving, cleaning and decontamination
  Preparation and packaging
  Sterilisation and
  Storage. [AII]

## Conclusion

We recommend all dental facilities to aim for excellence in IPC practices. COVID-19 pandemic had expedited rapid changes in many dental practices globally but these changes should be long-lasting with regular reviews for strategic revisions towards achieving staff and patient safety. A checklist for self-assessment is included in the guidelines to help in identifying gaps for improvement. The APSIC guideline not only gives timely guidance on safer practices at dental facilities during the COVID-19 pandemic but its continual implementation will certainly assist dental facilities to ensure appropriate safe practices are in place to mitigate healthcare associated infections.

### Endorsed by

1. Hong Kong Infection Control Nurses Association (HKICNA), Hong Kong
2. Infection Control Association of Singapore (ICAS), Singapore
3. Japanese Society for Infection Prevention and Control (JSIPC), Japan
4. Korean Society of Health-associated Infection Control (KOSHIC), South Korea
5. National Nosocomial Infection Group of Thailand, Thailand
6. Nursing Association for Prevention & Control of Infection, Thailand (ThaiNAPCI)
7. Philippine Hospital infection Control Society, (PHICS), Philippines
8. Infection Control Society of Taiwan (ICST)
9. Malaysian Society of Infection Control and Infectious Disease (MyICID), Malaysia
10. Indonesian Society of Infection Control (INASIC), Indonesia

## Data Availability

Yes; the APSIC Dental Infection Prevention and Control (IPC) Guidelines is available at the APSIC website (aspic-apac.org).
